# Three new species of *Tricimba* Lioy from the West Palaearctic region (Diptera, Chloropidae)

**DOI:** 10.3897/zookeys.558.6930

**Published:** 2016-02-01

**Authors:** Štěpán Kubík, Miroslav Barták, Hasan Civelek

**Affiliations:** 1 Czech University of Life Sciences, Faculty of Agrobiology, Food and Natural Resources, Department of Zoology and Fisheries, 165 21 Praha 6 - Suchdol, Czech Republic; 2 Department of Biology, Faculty of Sciences, Muğla Sıtkı Koçman Üniversitesi, 48000 Kötekli/Muğla, Turkey

**Keywords:** Diptera, Acalyptratae, Oscinellinae, taxonomy, new records, Corsica, Czech Republic, France, Portugal, Turkey

## Abstract

*Tricimba rudolfi* Kubík, **sp. n.** (Czech Republic, Portugal), *Tricimba chalupi* Kubík, **sp. n.** (Czech Republic), and *Tricimba dursuni* Kubík, **sp. n.** (Turkey) are described and illustrated. First records of *Tricimba kaplanae* Dely-Draskovits, 1983 from Corsica and *Tricimba hungarica* Dely-Draskovits, 1983 from Turkey are listed.

## Introduction

The genus *Tricimba* was erected by Lioy (1864). Species belonging to this genus may be distinguished from the remaining genera of the extant Palaearctic Oscinellinae by the presence of three or five more or less deeply impressed longitudinal grooves on the scutum, equipped with criss-cross arrangement of stiff micro-setulae. Fig. 19 in von Tschirnhaus and Hoffeins (2009) demonstrated such grooves in the fossil sister genus *Protoscinella* Hennig. Beschovski (1981) divided the genus *Tricimba* (only species from the Palaearctic Region) into three subgenera – *Tricimba* s. str., *Nartshukiella* Beschovski, and *Schumanniella* Beschovski, but those subgenera were not accepted by Ismay (1991) in the most thorough study of the genus *Tricimba*. With convincing arguments in that article he only defined species groups. Moreover Beschovski erroneously stated that the type species *Schumaniella setulosa* did not have two separate male cerci but instead both were fused to a mesolobus. However, a mesolobus is omitted in his figure of the genitalia, possibly as the cerci were damaged during dissection. M. von Tschirnhaus stated in his own material that the species has typical cerci similar to *Tricimba cincta* (Meigen, 1830). Dely-Draskovits (1983) revised the Palaearctic species of the genus. The larvae of several species are associated with fungi (Ševčík 2010) and several authors before him observed the same. Other species have been reared from rotting grasses and fruits (von Tschirnhaus 1982: 122) and *Tricimba sulcella* (Zetterstedt, 1848) developed in exposed dead snails (von Tschirnhaus 1992). The genus *Tricimba* is widely distributed in all faunal realms and it is the only extant genus within the Chloropidae which was identified already in Baltic and Dominican amber (Tertiary) (von Tschirnhaus and Hoffeins 2009). In the Palaearctic region 23 described valid species occur: *Tricimba albiseta* Dely-Draskovits, 1983, *Tricimba apicalis* (von Roser, 1840) *Tricimba bimarginata* Sabrosky, 1979, *Tricimba brachyptera* (Thalhammer, 1913), *Tricimba cincta* (Meigen, 1830), *Tricimba curvata* Sabrosky, 1961, *Tricimba freidbergi* Dely-Draskovits, 1983, *Tricimba heratica* Dely-Draskovits, 1983, *Tricimba humeralis* (Loew, 1858), *Tricimba hungarica* Dely-Draskovits, 1983, *Tricimba japonica* Dely-Draskovits, 1983, *Tricimba kaplanae* Dely-Draskovits, 1983, *Tricimba lineella* (Fallen, 1820), *Tricimba magna* Dely-Draskovits, 1983, *Tricimba meridiana* Dely-Draskovits, 1983, *Tricimba minima* (Vanschuytbroeck, 1945), *Tricimba paraalbiseta* Dely-Draskovits, 1983, *Tricimba parasetulosa* Dely-Draskovits, 1983, *Tricimba pulla* Dely-Draskovits, 1983, *Tricimba setulosa* (Becker, 1903), *Tricimba stigma* Kanmyia, 1983, *Tricimba submagna* Dely-Draskovits, 1983 and *Tricimba sulcella* (Zetterstedt, 1848). *Tricimba bimarginata* was added to the Palaearctic Region (Portugal) only recently by Ebejer and Andrade (2015). Further eight Palaearctic taxa are synonyms: *Tricimba aristolochiae*, *Tricimba delpinii*, *Tricimba flavipila*, *Tricimba fungicola*, *Tricimba maculifrons*, *Tricimba opacifrons*, *Tricimba punctifrons*, *Tricimba trisulcata* [and *spinigera*, a Nearctic syn. of *Tricimba lineella*]). (Duda 1932-1933; Sabrosky 1961; Beschovski 1981; Dely-Draskovits 1983; Kanmiya 1983; Deeming and Al-Dhafer 2012; Nartshuk 2013). Three additional species of this genus are added here.

## Material and methods

The studied material is deposited in the collections of the Czech University of Life Sciences, Prague (CULSP).

The genitalia were macerated in 10% KOH (24 hours, room temperature) and later stored together with the specimens on plastic tags and fixed with butyl-methacrylate copolymer of methyl-methacrylate, xylene. The genitalia were photographed by means of an Olympus E-41 digital camera mounted on an Olympus BX51 compound microscope and images were edited with the computer software Quick Foto micro 2.3 provided with Deep focus 3.1. Each image resulted usually from combining 7–15 layers. Images were improved by means of Adobe Photoshop and they served as models for outline of hand drawn illustrations; details were added by direct observing the genitalia. Individual species were photographed by means of a Nikon D300 digital camera mounted on an Nikon SMZ-U microscope and images were edited with the computer software NIS-Elements 3.0. Each image resulted usually from combining 15 layers. Images were improved by means of Adobe Photoshop. The morphological terms used here follow Merz and Haenni (2000). Abbreviations: f1,2,3 = fore, mid, hind femur, t1,2,3 = fore, mid, hind tibia. The length of the ocellar triangle was measured from the posterior margin of the posterior ocelli to the apex of the main part of the ocellar triangle. The head width was expressed as the distance of the widest points of the compound eyes. The head length was measured from the level of the posterior of the head horizontally to the level of the foremost extension of the anterior margin of frons or eye (depending what is most extending), excluding the antenna. The frons length was measured from posterior margin of posterior ocelli to the anterior margin of frons, the width across the dorsal posterior corners of the eye where the frons is the widest. In many species the number of orbital setae is difficult to judge as the setae are scarcely differentiated from the interfrontal setulae. The length of the scutum was measured from the level of the anterior margin to the base of scutellum, its width as the distance between the widest lateral points anterior of the wing base. All measurements (including body length) were taken from dry specimens (therefore the actual length may differ). The body length of males were measured from the antennal base to the hind end of the epandrium.

We were not able to compare the new species with all described species from the adjacent zoogeographical regions, Oriental, Afrotropical and Nearctic. Some Palaearctic *Tricimba* species are known also from tropical Africa (Deeming and Al-Dhafer 2012), the Oriental and/or Nearctic Regions: *bimarginata*, *cincta*, *humeralis*, *lineella*, *setosa*, *setulosa*, and *stigma*. The Afrotropical species *Tricimba africana* (l.c.) occurs also in South Yemen, belonging to the Afrotropics while North Yemen is Palaearctic, and Oriental species in India occur near to the Palaearctic Afghanistan.

## Systematic treatment

### Tricimba

Taxon classificationAnimaliaDipteraChloropidae

Lioy, 1864

Tricimba Lioy, 1864: 1125. Type species: *Oscinis lineella* Fallén, 1820: 8 by designation Enderlein 1911: 207.

#### Note.

The 15 generic synonyms of the genus were listed chronologically by Nartshuk (2012).

### Tricimba rudolfi

Taxon classificationAnimaliaDipteraChloropidae

Kubík sp. n.

http://zoobank.org/F8F5770C-CDB9-4535-8CE5-848F71224D9F

Figs 1 , 2 , 3

#### Holotype male.

**Czech Republic, Moravia**, Horní les u Lednice, 29.vi.1997, leg. R. Rozkošný. Holotype is in good condition.

#### Paratypes.

1 male, same data as the holotype, 1 male and 1 female: CZ Moravia, Podyjí NP, Nad Šobesem, Malaise trap, 2.vi.–2.vii.2003, leg. Š. Kubík, 1 male and 1 female, **Portugal**, Valhelhas, 500m, near river, SW+PT [= sweeping vegetation and yellow pan water traps], 40°24'10"N, 7°24'16"W, 16.–17.vii.2009, leg. M. Barták.

#### Distribution.

Czech Republic, Portugal

#### Date of occurrence.

June–July.

#### Diagnosis.

*Tricimba rudolfi* Kubík sp. n. is similar to *Tricimba lineella*. The main characters distinguishing these two species are as follows: *Tricimba rudolfi* has scutellum longer than wide, flat and triangular. Dorsal side has sharp margin and two rows of small pale setulae. Apical scutellar setae are divergent. *Tricimba lineella* has scutellum wider than long, more rounded and convex, without sharp margin on upper side. Apical scutellar setae convergent.

#### Description.

*Male*. Head wider than long. Frons about as wide as long, slightly concave, brownish black, brownish yellow on anterior 1/4, lateral margins almost parallel, anterior margin slightly produced anteriorly to anterior margin of eye. All setae and setulae pale. Frons with sparse setulae. Ocellar tubercle scarcely raised above level of remainder of frons. Ocellar triangle well developed, occupying 1/2 wide of frons posteriorly, extending almost 1/2 distance between anterior ocellus and anterior margin of frons, dusted with light grey microtoment. Ocellar setae small, upright and convergent, postocellar setae as long as ocellar setae, convergent. Outer vertical setae slightly longer than ocellar, inner vertical small. Altogether 6-7 thin and short orbital setae developed. Pedicel yellow. First flagellomere yellow, darkened on dorsal margin and base of arista. Arista thin, brown with pubescence as long as its basal diameter. Face yellow with keel between antennae, vibrissal angle projecting before margin of eye. Eye deeper than long, long axis nearly vertical. Gena broader than fore tibia, yellow, dusted, lower margin brownish yellow with two rows of pale setulae. Postgena dark, dusted, as broad as lower part of gena. Occiput dark brown, dusted, with one row of pale occipital setae. Proboscis brown. Palpus yellow, narrow, with pale yellow setulae.

**Figure 1.**
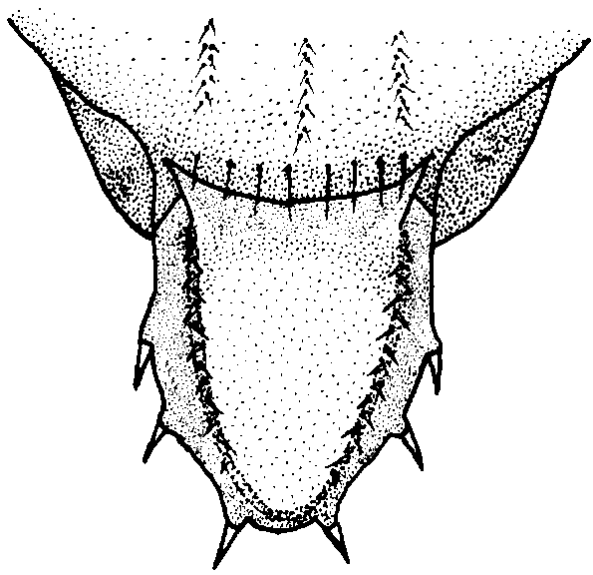
*Tricimba rudolfi* Kubík, sp. n. (holotype): scutellum dorsal view.

Scutum slightly longer than broad, dark brown and gray microtrichose, with 5 longitudinal deeply impressed grooves along acrostichal and dorsocentral lines and laterally from the latter ones. Notopleural setae 1+1. Anepisternum, katepisternum, and anepimeron dark brown and microtrichose except a shiny anterior lower margin of anepisternum and anterior margin of katepisternum. Scutellum (Fig. 1) longer than broad, flat, triangular, brown and dusted. Upper side with sharp margin and this with two rows of short pale setulae. Three pairs of short pale marginal setae each on a minute tubercle. Divergent apical pair slightly longer than lateral setae. Subscutellum developed, black, dusted dorsally and shining ventrally.

**Figure 2.**
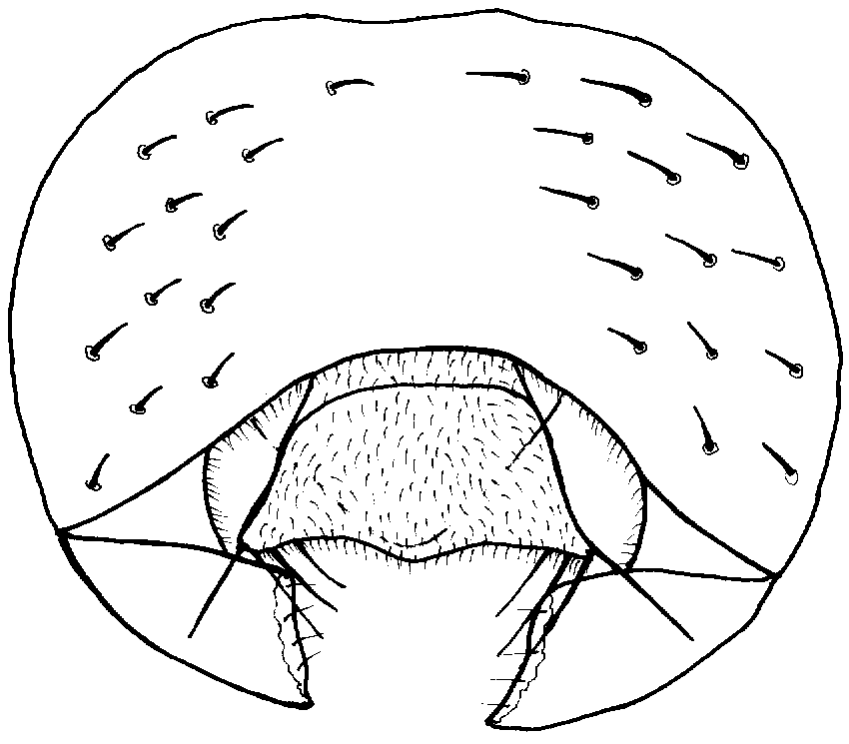
*Tricimba rudolfi* Kubík, sp. n. (holotype): epandrium posterior view.

Legs yellow, t3 with narrow brown band in middle. Wings hyaline with brown veins, R_2+3_, R_4+5_ and M_1+2_ almost parallel, second costal section as long as third costal section. Halter whitish yellow. Abdomen dark brown. Epandrium as in Fig. 2.

**Figure 3.**
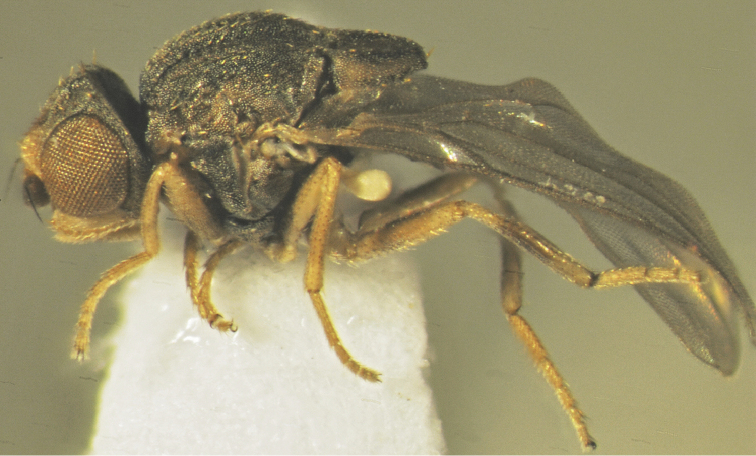
*Tricimba rudolfi* Kubík, sp. n. (holotype): body lateral view.

Length: 1.5–2 mm.

*Female*. Similar to male. Cercus dark brown with pale setulae.

#### Etymology.

named in honour of Professor Rudolf Rozkošný (Brno), collector of the holotype.

### Tricimba chalupi

Taxon classificationAnimaliaDipteraChloropidae

Kubík sp. n.

http://zoobank.org/47A10964-219F-4938-BA91-9606CCBA0C63

Figs 4 , 5

#### Holotype male.

**Czech Republic, Moravia**, Podyjí NP, Terasy mixed wood, 460 m, MT [= Malaise trap], 48°53'22"N, 15°50'18"E, 2.vii.–9.viii.2003, leg Š. Kubík and M. Barták. Holotype is in good condition but mid leg missing on left side.

#### Paratypes.

2 males, same data as holotype.

#### Distribution.

Czech Republic

#### Date of occurrence.

July–August.

#### Diagnosis.

**Figure 4.**
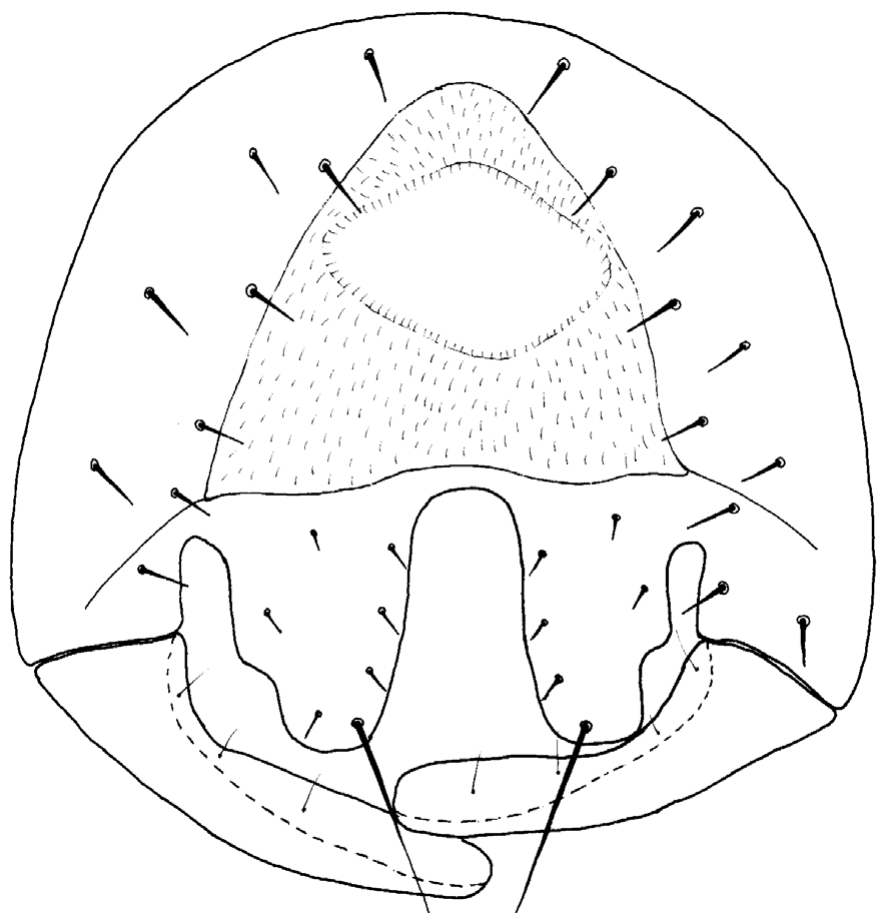
*Tricimba chalupi* Kubík sp. n. (holotype): epandrium posterior view.

*Tricimba chalupi* Kubík, sp. n. belongs to the group of thirteen Palaearctic very similar former *Nartshukiella* species which are difficult to separate. The main characters distinguishing this species are as follows: dark species with all setae and setulae black and with large bevelled cerci (Fig. 4).

**Figure 5.**
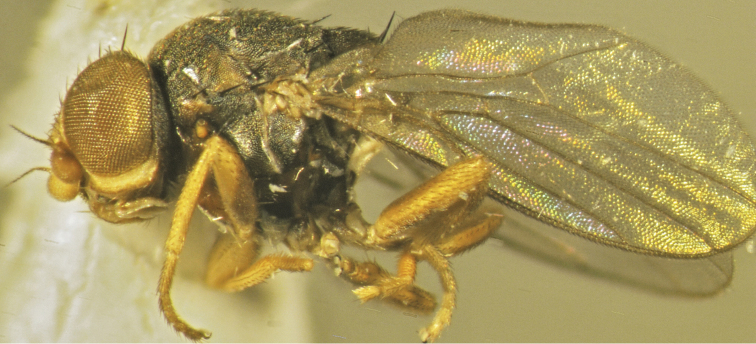
*Tricimba chalupi* Kubík, sp. n. (holotype): body lateral view.

#### Description.

*Male*. Head wider than long. Frons as wide as long, posterior half dark brown to black, dusted, anterior half brownish yellow, slightly concave, lateral margins parallel. All setae and setulae dark. Frons with irregular dark setulae, pale setulae occur only in front part of frons. Ocellar tubercle scarcely raised above level of remainder of frons. Ocellar triangle large, occupying more than half of posterior margin of frons, lateral margins slightly convex, main part extending more than halfway between anterior ocellus and anterior margin of frons, dusted with light grey microtoment, with one row of dark setulae along lateral margin. Ocellar setae as long as the distance between ocelli, upright and convergent, postocellar setae longer than ocellar setae, convergent. Outer vertical setae as long as postocellar setae, inner vertical smaller, as long as ocellar setae, 9–10 dark orbital setae, posterior five of them longer and stronger then the anterior ones. Antennae yellow, first flagellomere rounded. Arista thin, brown with pubescence of equal length as its basal diameter. Face yellow, vibrissal angle slightly projecting before anterior margin of eye. Eyes with scattered short ommatrichia, deeper than long, long axis vertical. Gena slightly broader than fore tibia in middle, yellow, dusted, with one row of pale long setulae. Postgena dark brown, dusted, slightly narrower than gena. Occiput dark and dusted. Proboscis and palpus yellow with pale setulae.

Scutum slightly longer than broad, black and dusted with gray microtrichosity, with three grooves of punctuations along acrostichal and dorsocentral lines, lateral groove not developed, 1+2 notopleural setae. Anepisternum black and dusted with gray microtrichosity, anepimeron similarly dusted as anepisternum, but posteroventrally with small shiny spot, katepisternum dark brown with dorsal half shiny and ventral gray dusted. Scutellum as long as broad, flat, rounded, black and dusted with gray microtrichosity, brownish yellow only on apical third. Three pairs of black marginal setae. Apical setae as long as half of scutellum and convergent, lateral short, one third as long than apical. Subscutellum developed, black, dusted dorsally, shining ventrally.

Legs yellow, f1-f3 with narrow brown band in middle. Wings hyaline with dark brown veins. Second costal section longer than third. Halter whitish yellow.

Epandrium as in Fig. 4.

Length: 2–2.5 mm

*Female*. Unknown.

#### Etymology.

Named in honour of our friend Tomáš Chalupa who popularized the National Park Podyjí.

#### Remarks.

This species may be identified with difficulties in the key by Dely-Draskovits (1983) because significant differences are only present in male genitalia. We propose to modify couplet 31 of the key as follows:

**Table:** 

31	Larger, body length 1.7–2.5 mm	**31a**
31a	Cerci short and rounded, body gray-brown, body length 1.7mm	***Tricimba sulcella* (Zetterstedt, 1818)**
31b	Cerci large and bevelled (Fig. 4), body black, body length 2-2.5mm	***Tricimba chalupi* Kubík, sp. n.**
32	Smaller, body length 1.4–1.5 mm	**33**

### Tricimba dursuni

Taxon classificationAnimaliaDipteraChloropidae

Kubík sp. n.

http://zoobank.org/1B61D35B-41DF-47A8-A681-015FA4AC6F08

Figs 6 , 7 , 10

#### Holotype male.

Turkey, Akyaka, pasture, 4m, 37°03'08.9"N, 28°20'17.4"E, 16.–22.ix.2012 leg. Barták and Kubík. Holotype is in good condition.

#### Paratypes.

19 males and 2 females, same data as the holotype.

#### Distribution.

Turkey.

#### Date of occurrence.

September.

#### Diagnosis.

**Figure 6.**
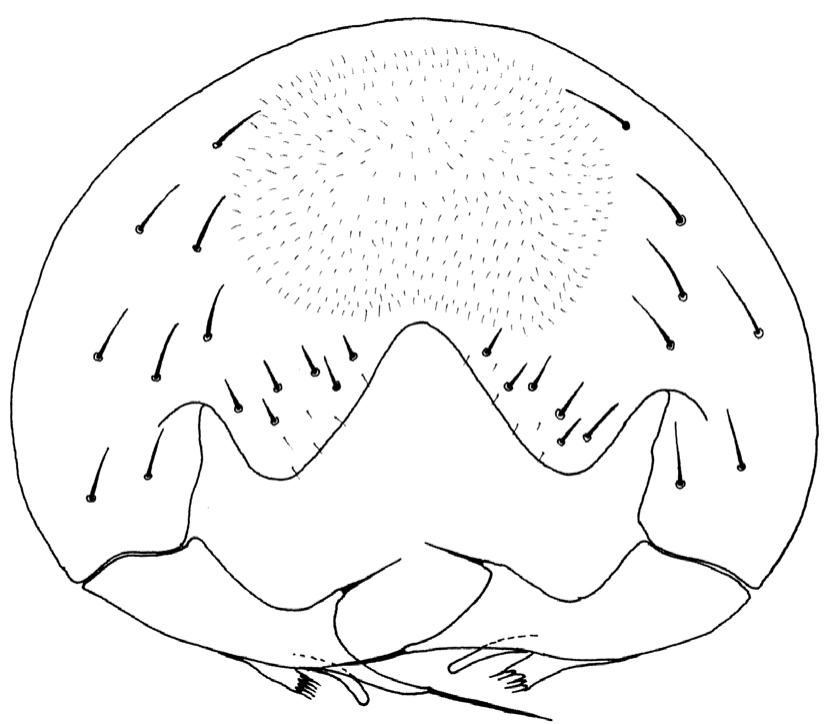
*Tricimba dursuni* Kubík, sp. n. (holotype): epandrium posterior view.

**Figure 7.**
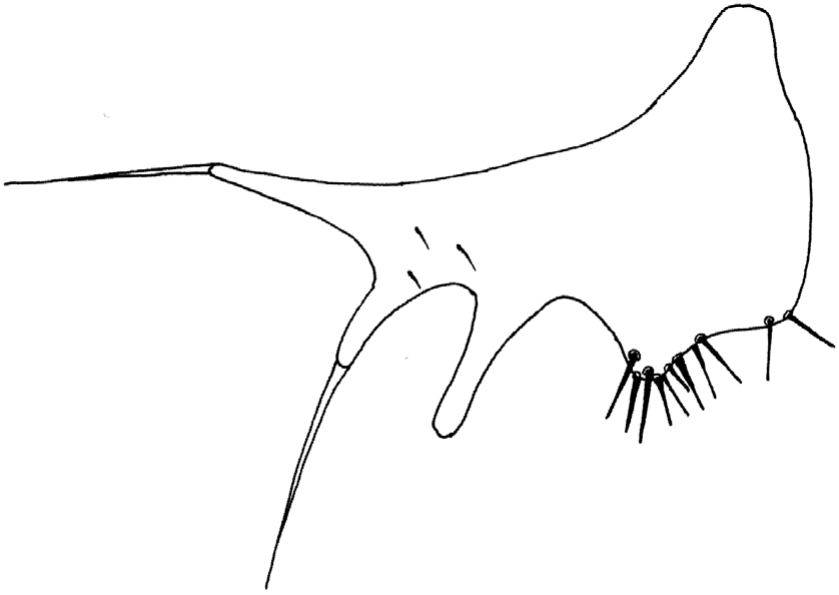
*Tricimba dursuni* Kubík, sp. n. (holotype): surstylus lateral view.

**Figure 8.**
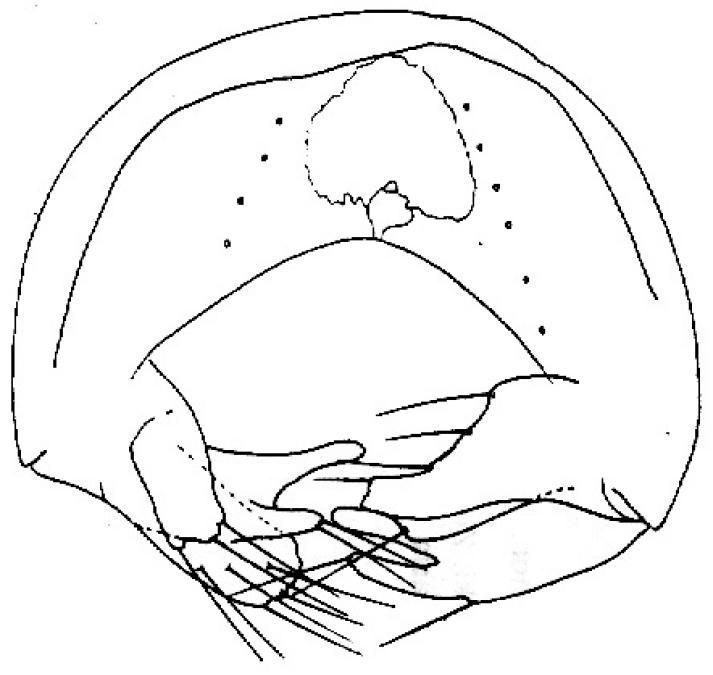
*Tricimba setulosa*: epandrium (after Beschovski 1981).

**Figure 9.**
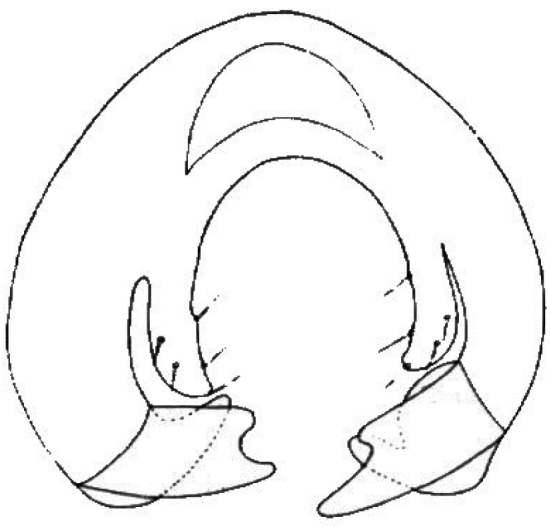
*Tricimba parasetulosa*: epandrium (after Dely Draskovits 1983).

*Tricimba dursuni* Kubík sp. n. belongs to the “extralimital taxon” (Ismay 1991: 303) together with *Tricimba setulosa* and *Tricimba parasetulosa* (phylogenetically first shifted here close to *setulosa*) in the Palaearctic Region. The main characters distinguishing these three species are as follows: frons brownish yellow on anterior half in *Tricimba dursuni*, but darker, yellowish brown on anterior 1/3 in *Tricimba setulosa* and 1/4 in *Tricimba parasetulosa*. *Tricimba dursuni* has pale setulae on scutum, both *Tricimba setulosa* and *Tricimba parasetulosa* have dark setulae. Genitalia strikingly differ in all these three species: *Tricimba dursuni* has surstylus with three long spurs and numerous black long setae on lower side (Figs 6–7), *Tricimba setulosa* and *Tricimba parasetulosa* have differently shaped surstylus (Figs 8–9).

**Figure 10.**
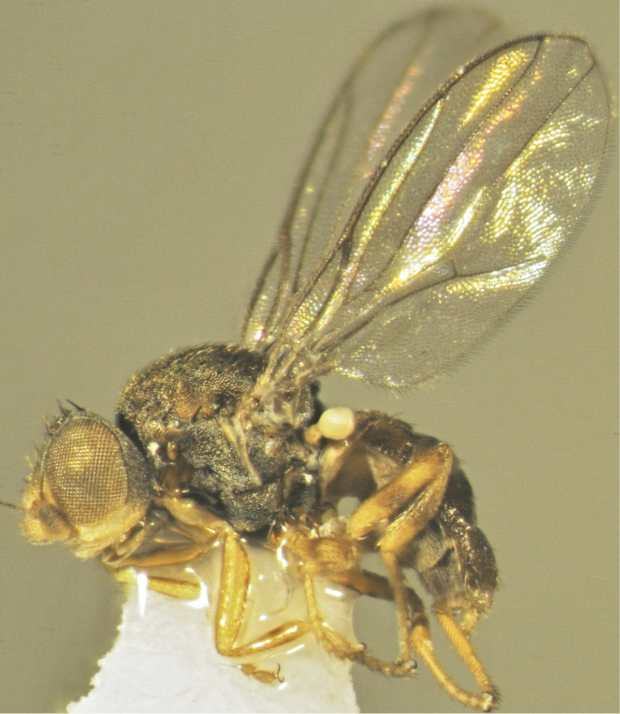
*Tricimba dursuni* Kubík, sp. n. (female paratype): body lateral view.

#### Description.

*Male*. Head longer than wide, as long as deep. Frons longer than wide, in front slightly concave, dark brown on posterior half, yellowish brown on anterior half, lateral margins parallel, anterior margin slightly produced before anterior margin of eye. All setae dark and setulae pale. Frons with sparse setulae. Ocellar tubercle scarcely raised above level of remainder of frons. Ocellar triangle well developed, occupying 2/3 width of frons posteriorly, extending almost ½ distance between anterior ocellus and anterior margin of frons, dusted with light gray microtrichosity. Ocellar setae small and convergent, postocellar seta as long as ocellar, convergent. Outer vertical setae slightly longer than inner vertical setae, 6-7 thin and small orbital setae, posterior four slightly longer than anterior. Pedicel yellow, first flagellomere yellow, darkened on dorsal margin and base of arista. Arista thin, brown, with pubescence subequal to its basal diameter. Face yellow, vibrissal angle not much projecting before margin of eye. Gena as broad as t3, yellow and dusted, lower margin with one row of pale setulae. Postgena ventrally dark, dusted, broader than gena. Occiput dark brown and dusted. Proboscis brown. Palpus yellow, narrow, with pale yellow setulae.

Scutum longer than wide, black, dusted with grey microtrichosity, with deeply impressed grooves and punctures. All setulae on scutum pale. Notopleural setae 1+2. Katepisternum, anepisternum and anepimeron dark brown and dusted. Scutellum longer than broad, flat, triangular, brown and dusted. Three pairs of black scutellar setae. Apical scutellar setae twice longer than lateral, black and convergent. Subscutellum developed, black, dusted dorsally, shining ventrally.

Legs yellow, t1, t2, t3, f2 and f3 with brown band in middle. Wings hyaline with dark veins, second costal section longer than third. Halter white.

Abdomen dark brown. Epandrium as in Figs 6–7.

Length: 1.5–2 mm.

*Female*. Similar to male.

#### Etymology.

Named in honour of Oktay Dursun, our colleague and dipterologist from Mugla University, Turkey.

#### Other examined material.

*Tricimba kaplanae* Dely-Draskovits, 1983: Corsica, 10 km W of Corte, 700m, Calacuccia env., 9.v.1993, 2 ♂, B. Mocek leg. This species has only been known from Israel. First record from the Corsica Isl.

*Tricimba hungarica* Dely-Draskovits, 1983: Turkey, Mugla, University campus, PT [= yellow and white pan water traps], 700m, 37°09'42"N, 28°22'21"E, 21.–24.ix.2012, 2♂, Barták and Kubík leg. This species has only been known from the Czech Republic, Hungary and Ukraine. First record from Turkey.

## Supplementary Material

XML Treatment for Tricimba

XML Treatment for Tricimba rudolfi

XML Treatment for Tricimba chalupi

XML Treatment for Tricimba dursuni
